# Hypocretin and brain β-amyloid peptide interactions in cognitive disorders and narcolepsy

**DOI:** 10.3389/fnagi.2014.00119

**Published:** 2014-06-11

**Authors:** Yves A. Dauvilliers, Sylvain Lehmann, Isabelle Jaussent, Audrey Gabelle

**Affiliations:** ^1^Sleep Unit, National Reference Network for Orphan Diseases (Narcolepsy, Hypersomnia, Kleine-Levin Syndrome), Department of Neurology, Gui de Chauliac Hospital, CHU MontpellierMontpellier, France; ^2^INSERM, U1061, Montpellier 1 UniversityMontpellier, France; ^3^Biochimie-Protéomique Clinique – IRMB – CCBHM -Inserm U1040 CHU MontpellierMontpellier, France; ^4^Clinical Research Memory Center Languedoc-Roussillon, Gui de Chauliac Hospital, CHU MontpellierMontpellier, France

**Keywords:** hypocretin, β-amyloid, Alzheimer's disease, cognition, sleep, CSF, tau

## Abstract

**Objective**: To examine relationships between cerebrospinal fluid (CSF) Alzheimer' disease (AD) biomarkers and hypocretin-1 levels in patients with cognitive abnormalities and hypocretin-deficient narcolepsy-cataplexy (NC), estimate diagnostic accuracy, and determine correlations with sleep disturbances.

**Background**: Sleep disturbances are frequent in AD. Interactions between brain β-amyloid (Aβ) aggregation and a wake-related neurotransmitter hypocretin have been reported in a mouse model of AD.

**Methods**: Ninety-one cognitive patients (37 AD, 16 mild cognitive impairment—MCI that converts to AD, 38 other dementias) and 15 elderly patients with NC were recruited. Patients were diagnosed blind to CSF results. CSF Aβ_42_, total tau, ptau_181_, and hypocretin-1 were measured. Sleep disturbances were assessed with questionnaires in 32 cognitive patients.

**Results**: Lower CSF Aβ_42_ but higher tau and P-tau levels were found in AD and MCI compared to other dementias. CSF hypocretin-1 levels were higher in patients with MCI due to AD compared to other dementias, with a similar tendency for patients with advanced AD. CSF hypocretin-1 was significantly and independently associated with AD/MCI due to AD, with an OR of 2.70 after full adjustment, exceeding that for Aβ_42_. Aβ_42_ correlated positively with hypocretin-1 levels in advanced stage AD. No association was found between sleep disturbances and CSF biomarkers. No patients with NC achieved pathological cutoffs for Aβ_42_, with respectively one and four patients with NC above tau and P-tau cutoffs and no correlations between hypocretin-1 and other biomarkers.

**Conclusions**: Our results suggest a pathophysiological relationship between Aβ_42_ and hypocretin-1 in the AD process, with higher CSF hypocretin-1 levels in early disease stages. Further longitudinal studies are needed to validate these biomarker interactions and to determine the cause-effect relationship and the role of wake/sleep behavior in amyloid plaque regulation.

## Introduction

Alzheimer's disease (AD) is a neurodegenerative disorder that is expected to become a disastrous worldwide epidemic within 40 years unless treatments to delay or halt the disease are developed. The pathological process is characterized by extracellular aggregation of β-amyloid peptides (Aβ) produced by amyloid precursor protein (APP) overexpression in the senile plaques and by intra-neuronal tau proteins in particular brain regions (Braak and Braak, 1996; Duyckaerts et al., 2009). However, the mechanisms of this pathological process remain only partly understood. In recent years, there is growing evidence that circadian and sleep disorders may play a causal role in the pathophysiology of AD (Kang et al., 2009; Roh et al., 2012). Sleep disturbances are frequently reported in AD, with both daytime sleepiness and nighttime awakenings (Dauvilliers, 2007; Ju et al., 2013; Spira et al., 2013). Non-specific factors associated with the ageing process and more specific ones, including disrupted sleep and circadian rhythm regulatory systems, are involved in sleep/wake disturbances (Montplaisir et al., 1995; Dauvilliers, 2007; Ju et al., 2013; Slats et al., 2013; Spira et al., 2013). Recently, interactions between the amyloid process and the sleep-wake switching system have been proposed. Brain interstitial fluid Aβ concentration increased during wakefulness and decreased during sleep in an APP transgenic mouse model of AD (Kang et al., 2009). Similar data were found in young healthy subjects, with peak CSF Aβ concentrations in the evening and lower concentrations overnight (Bateman et al., 2007; Kang et al., 2009). Further interactions were reported between brain Aβ aggregation and the wake-related neurotransmitter hypocretin/orexin in APP transgenic mice, with increased Aβ levels during hypocretin infusion and decreased levels under the effect of a hypocretin receptor antagonist (Kang et al., 2009). Significantly fewer hypocretin neurons were found in the post-mortem hypothalami of AD patients (Fronczek et al., 2012). However, several studies have reported a normal range of CSF hypocretin-1 concentration in AD patients (Mignot et al., 2002; Dauvilliers et al., 2003; Baumann et al., 2004; Friedman et al., 2007). One elegant study using an indwelling intrathecal catheter to collect hourly CSF samples in six patients with AD and six controls reported a correlation between hypocretin-1 and Aβ_42_, with no significant between-group differences in hypocretin-1 circadian rhythm amplitude (Slats et al., 2012). Moreover, lower CSF hypocretin-1 levels were found in dementia with Lewy bodies compared to AD and controls, with no associations between hypocretin-1 and Aβ_42_ concentrations (Wennstrom et al., 2012). However, CSF hypocretin-1 and tau levels were correlated in female non-demented controls (Wennstrom et al., 2012). Thus, the relationships between specific AD biomarkers and hypocretin-1 remain unclear, as well as relationships between sleep/wake states and brain Aβ fluctuations.

Our objective was to investigate the relationships between CSF Aβ_42_, tau, P-tau, and hypocretin-1 in a large population of patients with cognitive abnormalities, including patients at early and advanced stages of AD, patients affected with other dementias, and hypocretin-deficient patients with narcolepsy-cataplexy (NC).

## Materials and methods

### Patients

One hundred and six unrelated patients (60 males, 46 females; median age 69.3 years; range 32.1–89.5) participated. Patients with cognitive abnormalities comprised a small randomized sample from the Montpellier Cognitive biobank. Over 3000 baseline CSF samples were collected from patients attending the Montpellier Neurological and Clinical Research Memory Center (CMRR) for cognitive and behavioral disorders (official registration No. DC-2008-417). All patients underwent an extensive examination, including physical, neurological, and neuropsychological assessments, laboratory tests, and brain imaging. Diagnoses were performed blind to CSF results and by consensus by a multidisciplinary team of neurologists, geriatricians, and neuropsychologists. We selected 91 patients with cognitive complaints, including 37 with AD, 16 with amnesic mild cognitive impairment (MCI), and 38 with other dementias (DEM). Patients with AD met NINCDS-ADRDA clinical diagnostic criteria (McKhann et al., 1984, 2011). Patients with MCI initially met the usual criteria established by Petersen et al. (1999). All subjects that showed progressive cognitive decline to AD at a 2-to-7-year follow-up were considered MCI due to AD. The DEM group included frontotemporal lobar degeneration (FTLD) according to consensus criteria (McKhann et al., 2001), dementia with Lewy bodies (LBD) according to McKeith's criteria (McKeith et al., 1996; McKeith, 2006), and corticobasal degeneration according to Boeve et al.'s criteria (Boeve et al., 1999). Dementia severity was assessed using the Mini-Mental State Examination (MMSE) (Folstein et al., 1975).

Elderly patients with typical NC (American Academy of Sleep Medicine, 2005; Dauvilliers et al., 2007) who were followed at the Sleep Unit and with available CSF were included (*n* = 15; 9 males, 6 females; median age 65.6 years; range 54.0–86.4). Each patient had been clinically diagnosed with NC by a sleep specialist based on at least one night of polysomnographic recording followed by the Multiple Sleep Latency Test (MSLT), HLA-DQB1^*^0602 typing, and low CSF hypocretin-1 levels (<110 pg/mL) (Mignot et al., 2002; Dauvilliers et al., 2003, 2007). No patients with NC had cognitive abnormalities.

All patients gave their written informed consent to participate in the study. This study was approved by the regional ethics committee of the University of Montpellier Hospital.

### CSF samples and assays

CSF was obtained after a median duration of one month [interquartile range (IQR) 1–3 months] after diagnosis of cognitive abnormalities, in contrast to NC, with more than 30 years of delay between symptom onset and lumbar puncture in this elderly population. CSF was collected in polypropylene tubes under standardized conditions, preferably between 11:00 a.m. and 1:00 p.m. to minimize the influence of diurnal variation in CSF Aβ_42_ levels. CSF samples were transferred within 4 h after collection and centrifuged at 1000 g for 10 min at 4°C. A small amount of CSF was used for routine analyses, including total cell count, bacteriological exam, and total protein and glucose levels. CSF was aliquoted in polypropylene 1.5 mL tubes and stored at −80°C until further analysis. CSF Aβ_42_, total tau, and phospho-tau-181 (P-tau) were measured with Innotest® sandwich ELISA according to manufacturer's procedures (Innogenetics, Ghent, Belgium). The three biomarkers in each CSF sample were analyzed simultaneously. From these measurements, an Innotest® Amyloid Tau Index (IATI) was calculated for each patient. We used validated cutoffs for these biomarkers to clinically discriminate AD from normal aging and other neurologic disorders (Hulstaert et al., 1999).

Samples were rethawed for CSF hypocretin-1 measurement. CSF hypocretin-1 (orexin-A) was determined in duplicate for all subjects using I radioimmunoassay kits (Phoenix Peptide, Inc.) according to manufacturer's instructions. The detection limit was 10 pg/mL and intra-assay variability was <10%. CSF hypocretin-1 levels <110 pg/mL were considered low, intermediate from 110 to 200, and normal at >200. All values were back-referenced to Stanford reference samples (HHMI Stanford University Center for Narcolepsy, Palo Alto CA) (Mignot et al., 2002). The biological teams analyzed the CSF samples blind to clinical diagnosis.

### Sleep disturbances

To assess sleep disturbances, we conducted a short interview and administered a sleep questionnaire in a subgroup of 32 patients (*n* = 20 AD; *n* = 9 MCI due to AD; *n* = 3 DEM) within 2 years after CSF lumbar puncture. Patients were asked to rate their sleep duration based on bedtime, sleep latency, number of awakenings during the night, duration of awakenings, estimated total duration of nighttime sleep hours, and estimated total duration of wake time after sleep onset. The Epworth Sleepiness Scale was used to assess daytime sleepiness. Sleep questionnaires were completed by patients with the help of caregivers when required, especially for patients with AD and DEM.

### Statistical analysis

The sample is described in percentages for categorical variables and median and range for quantitative variables (age, CSF measurements), with distributions skewed using the Shapiro–Wilk statistic. Chi-square tests or Kruskall–Wallis tests were used to compare categorical and continuous characteristics between the four groups (AD, MCI due to AD, DEM, and NC). Two-group comparisons were performed using the Chi square (categorical variable) and Mann–Whitney Test (continuous variable). When comparisons were statistically significant, two-by-two comparisons were performed using the Bonferroni correction. Spearman's rank order correlations were applied to determine associations between two continuous variables. In order to determine which CSF measurements were independently associated with AD + MCI due to AD, measurements were entered simultaneously into a logistic regression model with potential confounders (e.g., age). Associations were thus quantified with odds ratios (OR) and their 95% confidence intervals (CI). Significance level was set at *p* < 0.05. Statistical analyses were performed using SAS, version 9.2 (SAS Institute, Cary, NC, USA).

## Results

### Demographic, clinical, and biological results by diagnostic category

Results on the demographic, clinical, and biological variables by diagnostic group are reported in Table 1. Significant group differences were observed for age, with patients with AD being the oldest and patients with NC the youngest. As expected, MMSE differed between groups, with scores for patients with either MCI due to AD or NC above the cutoff of 26. No gender effect was found on any CSF biomarker levels.

**Table 1:** **Sociodemographic, clinical, and biological variables for patients with different etiologies of cognitive impairment and patients with narcolepsy-cataplexy**.

	**AD**		**MCI due to AD**		**DEM**		**NC**		***p*-value**	***p*-value^a^**
	***N* = 37**		***N* = 16**		***N* = 38**		***N* = 15**			
Age (years)	72.30 [49.80–89.49]		73.20 [57.39–84.39]		66.84 [32.07–85.15]		65.59 [54.04–86.38]		0.03	
Gender										
Male	17	45.95	10	62.50	24	63.16	9	60.00	0.44	
Female	20	54.05	6	37.50	14	36.84	6	40.00		
MMSE	21.00 [2.00–25.00]		26.00 [26.00–30.00]		20.50 [9.00–30.00]		30.00 [30.00–30.00]		<0.0001	
MMSE										
<26	35	100.00	0	0.00	29	80.56	0	0.00	<0.0001	
≥26	0	0.00	14	100.00	7	19.44	15	100.00		
**CSF BIOMARKER MEASUREMENT**										
Aβ_42_ (pg/ml)	563.00 [232.00–1811.00]		593.00 [273.00–1505.00]		694.00 [306.00–1645.00]		947.00 [566.00–1332.00]		0.0005	0.02
Aβ_42_ (pg/ml)										
≤500	14	37.84	5	31.25	8	21.05	0	0.00	NA	NA
> 500	23	62.16	11	68.75	30	78.95	15	100.00		
Tau (pg/ml)	579.00 [283.00–2165.00]		706.50 [343.00–1200.00]		264.50 [104.00–1200.00]		215.00 [69.00–633.00]		<0.0001	<0.0001
Tau (pg/ml)										
≥cutoff^b^	26	70.27	11	68.75	12	31.58	1	6.67	<0.0001	
< cutoff	11	29.73	5	31.25	26	68.42	14	93.33		
P-tau (pg/ml)	79.00 [20.00–215.00]		106.50 [46.00–215.00]		41.50 [8.00–156.00]		41.00 [19.00–92.00]		<0.0001	<0.0001
P-tau (pg/ml)										
≥60	32	86.49	14	87.50	7	18.42	4	26.67	<0.0001	<0.0001
<60	5	13.51	2	12.50	31	81.58	11	73.33		
IATI index	0.62 [0.00–2.53]		0.60 [0.29–2.33]		1.28 [0.34–3.55]		1.76 [1.05–2.28]		<0.0001	<0.0001
IATI index										
<1	33	89.19	14	87.50	16	43.24	0	0.00	<0.0001	0.0009
≥1	4	10.81	2	12.50	21	56.76	15	100.00		
Hypocretin-1 (pg/ml)	451.00 [199.00–672.50]		503.75 [299.00–628.50]		386.00 [273.00–574.00]		34.00 [10.00–108.00]		0.03	0.06
Hypocretin-1 (pg/ml)										
<110	0	0.00	0	0.00	0	0.00	15	100.00	NA	NA
110–200	1	2.70	0	0.00	0	0.00	0	0.00		
≥200	36	97.30	16	100.00	38	100.00	0	0.00		

a *Adjusted for age*.

b *300 pg/ml (if aged 21–50 years), 450 (if aged 51–70 years), and 500 (if aged > 71 years)*.

*NA, Not applicable*.

**Figure 1:**
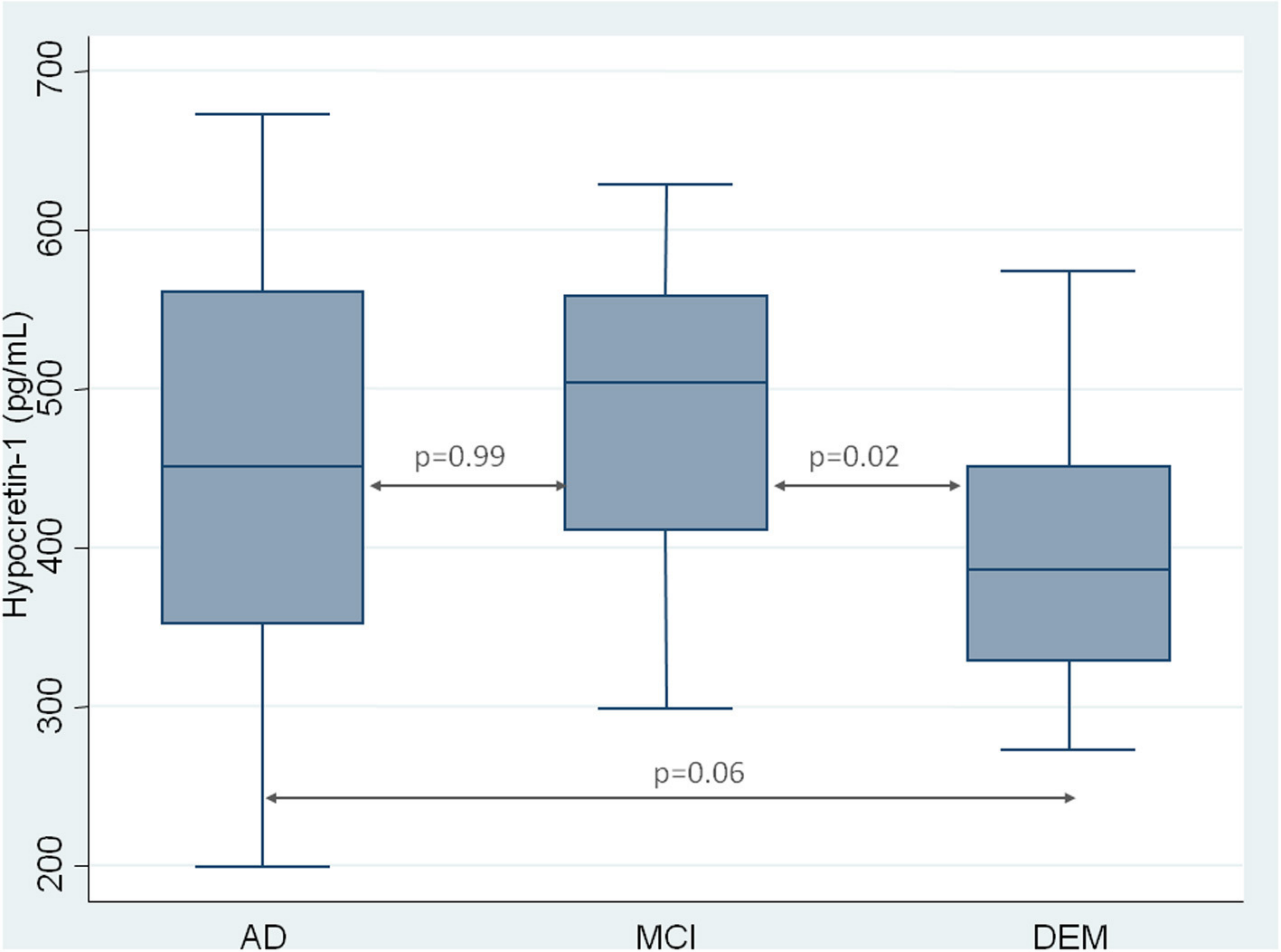
**CSF measurement of hypocretin-1 in patients with Alzheimer Disease (AD), Mild Cognitive Impairment (MCI), and with others Dementia (DEM)**. Results are shown as a box-whisker plot, with median and 25th quartile of CSF hypocretin-1 levels.

As expected, CSF hypocretin-1 levels differed between NC and other diagnostic groups (*p* < 0.0001 for all comparisons), with higher CSF hypocretin-1 levels in MCI due to AD, AD, and DEM groups compared to NC. Of the patients with cognitive abnormalities, CSF hypocretin-1 levels were higher in MCI due to AD compared to DEM (*p* = 0.02), with a similar tendency between AD and DEM (*p* = 0.06) (Figure 1). CSF biomarker levels, including Aβ_42_, tau, P-tau, and IATI, differed significantly between groups regardless of diagnostic category (continuous and categorically validated cutoffs) (Table 1). More precisely, CSF Aβ_42_ levels differed between the AD and NC (*p* < 0.0006) and between the MCI due to AD and NC groups (*p* = 0.013). Significant between-group differences were also found for CSF tau, P-tau, and IATI levels in the AD vs. DEM, AD vs. NC, MCI due to AD vs. DEM, and MCI due to AD vs. NC groups (*p* < 0.006 for all comparisons after Bonferroni correction). No patients with NC achieved pathological cutoffs for Aβ_42_ or IATI, with respectively one and four patients with NC above tau and P-tau cutoffs, all patients without cognitive abnormalities (Table 1). One patient with MCI due to AD had intermediate CSF hypocretin-1 levels, and no patients with either MCI due to AD, AD, or DEM had low hypocretin-1 levels.

### Comparisons between the AD and MCI due to AD and DEM groups

Because no CSF biomarker levels differed between the AD and MCI due to AD groups, and because follow-ups showed that all MCI developed AD, we decided to pool these two groups into one (AD+MCI-AD). The AD group included patients at early stage (MCI due to AD) and advanced stage (AD *per se*) of a similar disease. We further compared sociodemographic, clinical, and biological characteristics of patients with AD+MCI-AD and DEM (Table 2). A between-group age difference was found, with older patients in the AD+MCI-AD group. All CSF biomarker levels differed between groups, with lower Aβ_42_ and IATI and higher tau and P-tau levels in the AD+MCI-AD group. CSF hypocretin-1 levels also differed between groups, with higher levels in the AD+MCI-AD group (Table 2).

**Table 2:** **Comparison of sociodemographic, clinical, and biological variables between AD+MCI due to AD patients and patients with other etiology of dementia**.

	**DEM**		**AD + MCI due to AD**		***p*-value**	***p*-value^a^**
	***N* = 38**		***N* = 53**			
Age (years)	66.84 [32.07–85.15]		72.58 [49.80–89.49]		0.02	
Gender						
Male	24	63.16	27	50.94	0.25	
Female	14	36.84	26	49.06		
MMSE	20.50 [9.00–30.00]		23.00 [2.00–30.00]		0.09	
**CSF BIOMARKER MEASUREMENT**						
Aβ_42_ (pg/ml)	694.00 [306.00–1645.00]		568.00 [232.00–1811.00]		0.01	0.03
Aβ_42_ (pg/ml)						
≤500	8	21.05	19	35.85	0.13	0.19
>500	30	78.95	34	64.15		
Tau (pg/ml)	264.50 [104.00–1200.00]		588.00 [283.00–2165.00]		<0.0001	<0.0001
Tau (pg/ml)						
≥cutoff^b^	12	31.58	37	69.81	<0.0001	
< cutoff	26	68.42	16	30.19		
P-tau (pg/ml)	41.50 [8.00–156.00]		82.00 [20.00–215.00]		<0.0001	<0.0001
P-tau (pg/ml)						
≥60	7	18.42	46	86.79	<0.0001	<0.0001
<60	31	81.58	7	13.21		
IATI index	1.28 [0.34–3.55]		0.60 [0.00–2.53]		<0.0001	<0.0001
IATI Index						
<1	16	43.24	47	88.68	<0.0001	<0.0001
≥1	21	56.76	6	11.32		
Hypocretin-1 (pg/ml)	386.00 [273.00–574.00]		486.50 [199.00–672.50]		0.004	0.005
Hypocretin-1 (pg/ml)						
110–200	0	0.00	1	1.89	0.58	NA
≥200	38	100.00	52	98.11		

a *adjustment for age*.

b *300 pg/ml (if age between 21 and 50 years old), 450 (if age between 51 and 70 years old), and 500 (if age > 71 years old)*.

*NA, Not applicable*.

Two multivariate logistic regression models were conducted to determine the relative contribution of CSF biomarker measurements to the AD+MCI-AD diagnosis. The first model included hypocretin-1, tau, Aβ_42_, and age (Model 1), and the second included hypocretin-1, P-tau, Aβ_42_, and age (Model 2) (Table 3). After adjusting for these variables, hypocretin-1 levels remained significantly and independently associated with AD+MCI-AD, with an OR of 2.70 (95%*CI* = [1.37–5.32]) for Model 1 and an OR of 2.66 (95%*CI* = [1.35–5.23]) for Model 2, exceeding that for Aβ_42_ (Table 3).

**Table 3:** **Multivariate logistic regression models of CSF biomarker measurements associated with Alzheimer's Disease (AD)/Mild Cognitive impairment due to AD**.

**Model 1**		**Model 2**	
**Variables**	**OR [95%CI]^a^**	**Variables**	**OR [95%CI]^a^**
Hypocretin-1 (pg/ml)		Hypocretin-1 (pg/ml)	
OR for 110-unit increase	2.70 [1.37–5.32]	OR for 110-unit increase	2.66 [1.35–5.23]
Tau (pg/ml)		p-Tau (pg/ml)	
OR for 300-unit increase	4.24 [2.03–8.84]	OR for 60-unit increase	12.49 [3.61–43.23]
Aβ42 (pg/ml)		Aβ42 (pg/ml)	
OR for 500-unit increase	0.52 [0.20–1.33]	OR for 500-unit increase	0.44 [0.17–1.14]
Age (years)		Age (years)	
OR for 10-year increase	2.15 [1.05–4.40]	OR for 10-year increase	1.94 [0.96–3.92]

a *AD + MCI due to AD vs. DEM*.

### Correlations between CSF AD biomarkers and hypocretin-1

We found a positive correlation between hypocretin-1 levels and Aβ_42_ (*r* = 0.43, *p* = 0.001) and the IATI index (*r* = 0.42, *p* = 0.002) in the AD+MCI-AD group (*n* = 53). This can be explained by the patient group with advanced AD (AD: *r* = 0.45, *p* = 0.005; MCI due to AD: *r* = 0.38, *p* = 0.15). No significant correlations were found between hypocretin-1 and other biomarkers in the DEM group. In the whole group of 91 cognitive patients, the expected negative correlations between Aβ_42_ and tau (*r* = −0.3, *p* = 0.005) and between Aβ_42_ and P-tau (*r* = −0.22, *p* = 0.04) were found, but not for hypocretin-1 levels. Unexpectedly, we found positive correlations between CSF Aβ_42_ and tau (*r* = 0.69, *p* = 0.004) and P-tau (*r* = 0.70, *p* = 0.004) in patients with NC, but not for hypocretin-1 levels.

### Associations between CSF biomarkers and sleep disturbances

Sleep disturbances were assessed by clinical interview and questionnaires in a limited population sample (*n* = 32) with the help of caregivers when required. The mean MMSE for the 20 AD patients who completed sleep questionnaires is 19.9 [14–24], and 21.6 [14–26] for the 3 DEM patients. No associations were found between sleep disturbances (wake time after sleep onset, total sleep time, and Epworth Sleepiness Scale) and CSF biomarkers (Aβ_42_, tau, P-tau, and hypocretin-1) in either the whole assessed population or in the AD+MCI-AD group only (*n* = 29).

## Discussion

We investigated the relationships between CSF Aβ_42_, tau, P-tau, and hypocretin-1 levels in a large clinically based cohort of patients with different etiologies of cognitive abnormalities and hypocretin-deficient narcoleptic patients. Higher CSF hypocretin-1 levels were found in all demented patients compared to control narcoleptic patients without cognitive abnormalities. More interestingly, CSF hypocretin-1 levels were higher in patients with MCI due to AD compared to patients with other etiologies of dementia, with a similar tendency for advanced AD. Because all patients with MCI in our cohort evolved toward the AD process, we pooled the AD patients into an early and advanced stage AD group. Multivariate analysis results suggest that CSF hypocretin-1 concentrations could contribute to the AD diagnosis, as significant levels were found in early stage AD. Moreover, a positive correlation between Aβ_42_ and hypocretin-1 levels was observed in advanced AD, with no association with sleep disturbances measured by sleep questionnaires only. Furthermore, no association was found between CSF, tau, or P-tau and hypocretin-1 levels.

CSF biomarkers are widely used for diagnosing AD in atypical clinical forms and for differential and early diagnosis (Bateman et al., 2007; Blennow and Zetterberg, 2009; Mattsson et al., 2009; Gabelle et al., 2011). A major contribution of our study is that it underscores the greater contribution of using hypocretin-1 compared to Aβ_42_ to differentiate an AD + MCI due to AD diagnosis from other dementia diagnoses. Although our results confirmed that common amyloid and tau CSF biomarkers help diagnose AD even at early stages, more interestingly, hypocretin-1 is significantly and independently associated with this group of patients, with an OR of 2.70 after full adjustment. The diagnostic interest of hypocretin-1 is also highlighted by the fact that its CSF concentrations differed significantly between DEM and MCI patients that converted into AD. This indicates that hypocretin-1 could be used for early detection of AD. The underlying rationale is that amyloid pathology is believed to be present very early in the development of the disease, and Aβ_42_ and hypocretin were correlated in AD. Previous studies have shown normal CSF hypocretin-1 levels in AD (Mignot et al., 2002; Dauvilliers et al., 2003), and normal or low levels in LBD (Baumann et al., 2004; Wennstrom et al., 2012). However, to our knowledge, none of these studies compared patients at different stages of the AD process, and none specifically included patients affected with MCI. Between-group comparisons of CSF hypocretin-1 levels in patients with different etiologies of cognitive abnormalities are rare, and pathological cutoffs for CSF hypocretin-1 levels have been validated for narcolepsy only (Mignot et al., 2002; Dauvilliers et al., 2003, 2007). One study reported a 40% reduction in the total number of hypocretin neurons in AD (Fronczek et al., 2012), but no correlation has yet been reported between CSF hypocretin-1 levels and the number of hypocretin neurons in humans, as surviving neurons might compensate for lost neurons by increasing hypocretin synthesis.

Another striking finding of the present study is the positive correlation between hypocretin-1 and Aβ_42_ levels in AD, but not in other dementias or in NC. Because the patients with MCI were seen regularly at the CMRR, with follow-up revealing conversion to AD, we were able to determine largely pronounced brain β-amyloid and hypocretin-1 interactions in advanced stage AD. Using *in vivo* microdialysis in APP transgenic mice, a seminal study highlighted significant interactions between brain extracellular accumulations of Aβ peptide, wakefulness, and hypocretin-1 (Kang et al., 2009). The same authors recently demonstrated a strong relationship between disrupted sleep-wake states and increased wakefulness, loss of circadian Aβ fluctuation, and neuronal activity (through lactate metabolism) in APP transgenic mice only after amyloid plaque formation, and changes were prevented by Aβ_42_ active immunization (Roh et al., 2012).

Human studies of CSF samples collected via lumbar catheters in young healthy volunteers have revealed peak CSF Aβ concentrations in the evening and lower concentrations overnight (Bateman et al., 2007; Kang et al., 2009). A slight correlation between Aβ_42_ and hypocretin-1 levels was recently reported in 6 patients with AD and 6 healthy controls, with no between-group difference in CSF hypocretin-1 levels and its circadian amplitude (Slats et al., 2012). In contrast, no association between Aβ_42_ and hypocretin-1 was reported in either control subjects or patients with AD in a larger study, but with low hypocretin-1 levels in 9 female patients with LBD (Wennstrom et al., 2012). In young patients with AD carrying presenilin mutations, CSF Aβ fluctuation disappeared after amyloid plaque formation (Roh et al., 2012). These varying results on relationships between brain Aβ and hypocretin-1 levels across human studies may be due to small sample sizes, differences in methodology and population characteristics, and the influence of uncontrolled factors such as sleep-wake states.

Sleep-wake behavior is a fundamental brain function that is substantially associated with cognition and synaptic plasticity (Tononi and Cirelli, 2006). A recent study in mice reported that sleep is critical for ensuring metabolic homeostasis: large increases in the cortical interstitial space during sleep result in convective exchange between CSF and the interstitial fluid (Xie et al., 2013). The restorative function of sleep may therefore be related to increased clearance of potentially neurotoxic degradation products of neuronal activity that accumulate in the awake brain, such as Aβ (Xie et al., 2013). Daytime wake and nighttime sleep fragmentation have been frequently reported in AD, but with an unclear biological basis (Montplaisir et al., 1995; Dauvilliers et al., 2003; Kang et al., 2009). Because hypocretin is a major wake-related neurotransmitter (de Lecea et al., 1998), few studies have focused on its impact on wakefulness consolidation in either patients with AD (Friedman et al., 2007) or elderly subjects (Ju et al., 2013; Spira et al., 2013). Using wrist actigraphy recording, one study reported increased wake fragmentation in patients with lower CSF hypocretin-1 levels (Friedman et al., 2007). A recent study revealed an association between CSF Aβ_42_ and poor sleep quality, but not sleep quantity, using actigraphy in cognitively normal subjects potentially in the preclinical stage of AD (i.e., 50% having parental history of late-onset AD) (Ju et al., 2013). Recently, a self-reported assessment of sleep parameters in community-dwelling older adults showed that both shorter sleep duration and poorer sleep quality were associated with greater Aβ burden, measured by Pittsburgh compound B positron emission tomography (Spira et al., 2013). Unfortunately, as in the previous study, our protocol did not include actigraphy or polysomnography recordings. To determine the impact of sleep/wake states on the interaction between CSF Aβ_42_ and hypocretin-1 levels, we determined the presence of sleep disturbances in a limited population sample using clinical interviews and questionnaires with patients, with caregiver help when required. We found no associations between total sleep time, wake time after sleep onset, daytime sleepiness, and any CSF biomarkers. Further studies using objective tools to assess sleep disturbances in patients with AD are recommended as clinical interviews have limited value to assess this condition.

The degeneration of key sleep and wake regulatory systems in AD, which is responsible for a marked increase in nighttime wakefulness and daytime sleepiness, may interfere with neuronal metabolism *per se* and ultimately lead to Aβ aggregation. Interestingly in AD, the decrease in CSF Aβ_42_ associated with its aggregation in amyloid plaques is also correlated with other Aβ isoforms, including Aβ_40_ and soluble APPs, which are released mainly during physiological synaptic plasticity (Cole and Vassar, 2008; Gabelle et al., 2010). It is therefore possible that several factors believed to have a trophic function may play a role in this pathophysiology, with potential relationships with sleep-wake states (Friedman et al., 2007; Cole and Vassar, 2008; Xie et al., 2013). Although the process is largely unclear, amyloid plaque formation, a pathological hallmark of AD, may be modulated by the endogenous hypocretin signal.

Another limitation of the present study is the absence of healthy controls. However it was difficult to construct an appropriate control group of cognitively normal elderly adults with CSF lumbar puncture. We used CSF samples from elderly patients with NC characterized by daytime sleepiness, fragmented nighttime sleep, cataplexy, and particularly hypocretin deficiency. As expected, CSF Aβ_42_, tau, P-tau, and IATI levels differed significantly between AD, MCI due to AD, and NC groups. However, patients with NC with low CSF hypocretin-1 levels did not present low Aβ_42_ levels. Therefore, the positive correlation between CSF Aβ_42_ and hypocretin-1 levels appears to be specific to the AD process. However, our results in NC contrasted with a previous case report of a hypocretin-deficient young girl with NC with low CSF Aβ_42_ who had been vaccinated for pandemic H1N1 (Kallweit et al., 2012). Unexpectedly, we also found positive correlations between CSF Aβ_42_ and tau and P-tau levels in patients with NC with no cognitive abnormalities, but no relationship was found with hypocretin-1. These findings shed light on the complex relationship between these biomarkers and various underlying conditions.

To conclude, we examined CSF Aβ_42_, tau, P-tau, and hypocretin-1 levels in a large clinically based population of patients with cognitive abnormalities. Results indicate that significantly higher CSF hypocretin-1 concentrations in early stage AD could contribute to the AD diagnosis. These results also suggest a pathophysiological relationship between a key wake-related neurotransmitter (hypocretin-1) and brain accumulation of β-amyloid peptides in the AD process. Further longitudinal studies are required to validate these biomarker interactions and to determine the cause-effect relationship and the role of wake/sleep behavior in the regulation of amyloid plaque formation.

### Conflict of interest statement

The authors declare that the research was conducted in the absence of any commercial or financial relationships that could be construed as a potential conflict of interest.
